# Protected State Transfer via an Approximate Quantum Adder

**DOI:** 10.1038/s41598-017-06425-3

**Published:** 2017-07-31

**Authors:** G. Gatti, D. Barberena, M. Sanz, E. Solano

**Affiliations:** 1 Departamento de Ciencias, Sección Física, Pontificia Universidad Católica del Perú, Apartado, 1761 Lima Peru; 2 Department of Physical Chemistry, University of the Basque Country UPV/EHU, Apartado 644, E-48080 Bilbao, Spain; 3 IKERBASQUE, Basque Foundation for Science, Maria Diaz de Haro 3, 48011 Bilbao, Spain

## Abstract

We propose a decoherence protected protocol for sending single photon quantum states through depolarizing channels. This protocol is implemented via an approximate quantum adder engineered through spontaneous parametric down converters, and shows higher success probability than distilled quantum teleportation protocols for distances below a threshold depending on the properties of the channel.

## Introduction

Although decoherence has been described^1, 2^, measured^3, 4^, simulated^5–7^ and used for different quantum tasks^8–10^, it is often considered as one of the main setbacks of experimental setups based on quantum mechanics. This is evident in quantum computing, quantum simulations and quantum communication, which has triggered a race to find decoherence-resistant states and protocols, starting in the mid 1990s. Shor proposed one of the first quantum error correction codes for quantum computing^11^, closely followed by the quantum distillation protocol for teleportation in noisy channels, proposed by Bennet *et al*. in the framework of quantum communication^12^. The latter method is based on *distilling* a few high fidelity Bell pairs from several low fidelity copies. Later, decoherence slowdown through quantum feedback was proposed by Vitali *et al*.^13^, and the usage of noiseless (zero decoherence) subspaces was proposed by Zanardi *et al*.^14^. Quantum error correction codes based on topologically ordered states, such as a spin-1/2 honeycomb lattice, have been proposed^15, 16^ and experimentally demonstrated^17^. Additionally, methods making use of weak measurements have emerged to protect quantum states from decoherence^18, 19^. Recently, a cavity state transfer protocol assisted by temporal modes in a tailored waveguide was proposed in ref. 20. In all of these examples, we can distinguish error correction from decoherence protection methods. Within the latter group, quantum optics is a natural choice for researching protected state transmission against decoherence.

In the early 1990s, interference between coherently pumped down-converters emerged as a powerful tool in quantum optics. Particularly, the second-order spontaneous parametric down-conversion interference device (SPDC interference)^21, 22^ was proven to be key in fundamentals of quantum mechanics due to its nonlocality^23–25^. A variant of this array was exploited for ghost imaging without requiring coincidences^26^.

In the search for decoherence-protected systems, we will link SPDC interferometers with the theoretical construction of quantum adders^27^. A quantum adder is defined as a transformation in which the output is a superposition of two arbitrary unknown input states, previously codified in two different Hilbert spaces^27, 28^. This transformation is forbidden by quantum mechanics, but it can still be achieved by postselection for states which are nonorthogonal to a reference state^29^. Despite the experimental realization, no practical application has been found for probabilistic quantum adders yet.

In this Article, we propose a protocol for sending single photon quantum states protected against depolarizing channels. The setup is a direct application of a probabilistic quantum adder based on a second order SPDC interferometer. Our protocol consists in encoding a qubit, initially written in the polarization of a single photon, into the Fock space of two different paths. This encoded qubit is sent through decoherence channels and its original information is afterwards reconstructed by means of another nonlinear crystal. Our success probability for sending a qubit is not only higher than directly sending the state, but also than that obtained with distilled quantum teleportation for distances below a threshold depending on the properties of the channel.

## State preparation via quantum adder

A quantum adder^27^ consists in a quantum state transformation given by $|\alpha \rangle \otimes |{\psi }_{B}\rangle |{\psi }_{A}\rangle \to |\alpha ^{\prime} \rangle \otimes$ $|\chi \rangle R(|{\psi }_{A}\rangle +|{\psi }_{B}\rangle )$, where |*ψ*_*A*_〉 and |*ψ*_*B*_〉 are arbitrary states initially in two different Hilbert spaces (we choose qubits), *R* is a normalization constant, |*χ*〉 is a state that may also depend on the input states, and |*α*〉 and |*α*′〉 are ancillas. This quantum adder is forbidden by the unitarity condition of quantum mechanics^27, 28^.

In our setup, we construct a probabilistic generalized kind of quantum adder using spontanous parametric down-conversion. In our version of the quantum adder, we will not only add states, but also add them in different linear combinations. For the sake of simplicity, we fix one of these states. The type of linear combination is controlled by a parameter *f*, which may depend on any of the initial states. This transformation is

1 $$|\alpha \rangle \otimes |\chi \rangle |{\psi }_{A}\rangle \to |\alpha ^{\prime} \rangle \otimes |\chi ^{\prime} \rangle R(|{\psi }_{A}\rangle +f|{\psi }_{B}\rangle ),$$

where |*ψ*_*B*_〉 is a fixed state agreed beforehand.

**Figure 1:**
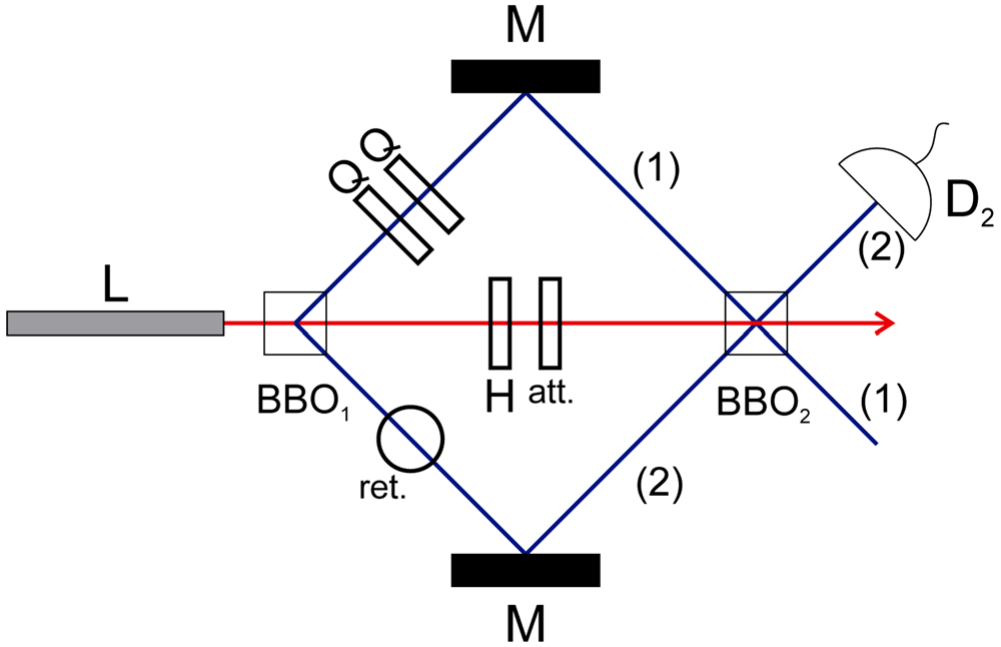
A coherent laser (red line) pumps two type-1 beta-barium-borate crystals (*BBO*), which, with probability amplitude *g*, emit pairs of single photons with polarization *V* in the specified paths (blue lines), via spontaneous parametric down-conversion (SPDC). The paths of the photons emitted by BBO_1_ are aligned with mirrors so that they match with the paths that BBO_2_ emissions (with the same frequency) would follow. *Q* and *H* are quarter and half waveplates, respectively. *ret*. is a retarder, *att*. is an intensity attenuator, *M* are mirrors, and *D*_2_ is a detector in path (2). Detections after BBO_2_ show interference between probability amplitudes of pairs of photons emitted in BBO_1_ and BBO_2_. Consequently, this is not interference between paths (1) and (2).

To achieve this, let us consider a pumped non-linear crystal (BBO_1_) where the paths of twin photons generated by spontaneous parametric down-conversion are aligned into a second crystal (BBO_2_) (see Fig. 1). As aforementioned, our setup is inspired in previous SPDC interferometers^21, 23^.

When twin photons are detected after BBO_2_, it is indistinguishable which BBO made the emission, and the uncertainty of the time of emission is sufficiently large to allow interference between both possibilities.

The two quarter-wave plates (*Q*) are sufficient to rotate the polarization of path (1) photons in one of our emission possibilities, BBO_1_, into an arbitrary chosen polarization |*ψ*_*A*_〉 = *a*_1_|*H*〉 + *a*_2_|*V*〉^30^. Without loss of generality, we choose a_2_ to be real. The retarder (*ret*.) compensates for any extra phase introduced by this rotation and by any path difference in the experiment, and is set to add a relative phase *ϕ* between the coherent laser and the photons. The half-wave plate (*H*) is set to rotate the pump beam, initially in horizontal polarization, into an arbitrary chosen polarization |*χ*〉 = *b*_1_|*H*〉 + *b*_2_|*V*〉, with *b*_1_ and *b*_2_ real, while the attenuator (*att*.) is set to reduce the intensity of the coherent laser by a factor of *A*, with 0 ≤ *A *≤ 1. Note that our protocol, after BBO_2_, involves interference between the probability amplitudes of emitted photons by BBO_1_ and BBO_2_. Therefore, we are not dealing with interference between paths (1) and (2).

Let us address this in a more formal manner. The initial state of the system, before BBO_1_, is |*α*〉_0*H*_|*0*〉_0*V*_(|0, 0〉_1_ |0, 0〉_2_), where |*α*〉_0*H*_|0〉_0*V*_ indicates that the laser pump has a horizontally polarized coherent state |*α*〉 and a vertically polarized coherent state |0〉. Additionally, |*n*_*H*_,*n*_*Vx*_〉 indicates that there are *n*_*H*_ (*n*_*V*_) horizontally (vertically) polarized photons in path *x*, with *x* = 1, 2. Note that we are not using a two-level description for the single-photon polarization, since we will have zero-photon states in our description. We will, however, return to the two-level description of polarization later on.

For the interaction between our system and the BBOs, we use the effective Hamiltonian ${H}_{{\rm{BBO}}}=$ $g^{\prime} ({\hat{a}}_{0H}{\hat{a}}_{1V}^{\dagger }{\hat{a}}_{2V}^{\dagger }+{\hat{a}}_{0H}^{\dagger }{\hat{a}}_{1V}{\hat{a}}_{2V})$ ^31^, where *â*_*xy*_ is the annihilation operator for path index *x* (0, 1 or 2) and polarization *y* (H or V), and ${\hat{a}}_{x\,y}^{\dagger }$ its respective creation operator. *g*′ is a crystal-dependent constant, which we assume to be real without loss of generality. We define $g=-\frac{g^{\prime} t}{\hslash }$, where *t* is the interaction time for the specific single-photon paths chosen. Note that *g* is small because *t* is of the order of the single-photon coherence length divided by the speed of light. We also consider *α* ≪ *g* ^−1^ for all coherent states *α* considered in this setup, to allow us to keep only low orders.

Right before BBO_2_, up to order *g* ^3^ in probability, we have the quantum state

2 $${|{e}^{i\varphi }A\alpha {b}_{1}\rangle}_{0H}{|{e}^{i\varphi }A\alpha {b}_{2}\rangle}_{0V}\otimes [(1-\frac{{g}^{2}|\alpha {|}^{2}}{2}){|0,0\rangle}_{1}{|0,0\rangle}_{2}+ig\alpha ({a}_{1}{|1,0\rangle}_{1}+{a}_{2}{|0,1\rangle}_{1}){|0,1\rangle}_{2}],$$

and after BBO_2_, to same order, we have the state

$$\begin{array}{c}{|{e}^{i\varphi }A\alpha {b}_{1}\rangle}_{0H}{|{e}^{i\varphi }A\alpha {b}_{2}\rangle}_{0V}\otimes [1-\frac{{g}^{2}|\alpha {|}^{2}}{2}(1+{A}^{2}|{b}_{1}{|}^{2}+2{e}^{-i\varphi }{a}_{2}{b}_{1}A){|0,0\rangle}_{1}{|0,0\rangle}_{2}ig\alpha \\ \quad \quad \times ({a}_{1}{|1,0\rangle}_{1}+({a}_{2}+{e}^{i\varphi }A{b}_{1}){|0,1\rangle}_{1}){|0,1\rangle}_{2}].\end{array}$$

Note that some states of amplitude of order *g* ^2^ are left in the expression, while other terms of the same amplitude order are eliminated. This is because some of these terms, like the *g* ^2^ in $1-\frac{{g}^{2}|\alpha {|}^{2}}{2}$, contribute to probability in order *g* ^2^, while other amplitude terms, like a simple *g* ^2^, contribute in order *g* ^4^. The states eliminated are guaranteed to never have an amplitude with probability contribution of order lower than *g* ^4^. A two photon mode, for instance, will always be of probability order *g* ^4^ or higher if the initial state only consists of a pump beam, even when several BBOs are used.

If we now placed a detector in paths (1) or (2) just before BBO_2_, the system state whenever a photon is received could be written using the {|*H*〉, |*V*〉} notation for polarization as

3 $${|{e}^{i\varphi }A\alpha {b}_{1}\rangle }_{0H}{|{e}^{i\varphi }A\alpha {b}_{2}\rangle }_{0V}\otimes ({a}_{1}{|H\rangle }_{1P}+{a}_{2}{|V\rangle }_{1P}){|V\rangle }_{2P},$$

since there would be no zero-photon states. If we instead placed the detector after BBO_2_, the state upon detection could be written as

4 $${|{e}^{i\varphi }A\alpha {b}_{1}\rangle }_{0H}{|{e}^{i\varphi }A\alpha {b}_{2}\rangle }_{0V}\otimes R({a}_{1}{|H\rangle }_{1P}+({a}_{2}+{e}^{i\varphi }A{b}_{1}){|V\rangle }_{1P}){|V\rangle }_{2P},$$

where $$R={(|{a}_{1}{|}^{2}+|{a}_{2}+{e}^{i\varphi }A{b}_{1}{|}^{2})}^{-1}$$.

Therefore, detected states before BBO_2_ can be mapped via our probabilistic quantum adder into detected states after BBO_2_. This effective transformation is given by Eq. (1) and is shown by considering the state |*ψ*_*A*_〉 = *a*_1_|*H*〉 + *a*_2_|*V*〉 in Eq. (3) and the (fixed) state |*ψ*_*B*_〉 = |*V*〉. These states appear added in a linear combination in Eq. (4), under a factor of $f={e}^{i\varphi }A{b}_{1}$, which depends on some of the states in Eq. (3).

## Protected state transfer

In this section, we use the previous setup for a quantum state transfer protocol protected against decoherence. Alice wants to send an arbitrary qubit |*ψ*_*A*_〉 = *a*_1_|*H*〉 + *a*_2_|*V*〉 to Bob and she creates this state in our previous setup by using the two *Q* waveplates. Alice sets *b*_1_ = 1, *A* = *a*_2_ and *ϕ* = *π* with her *H* waveplate, attenuator, and retarder. After BBO_2_, the system state is

5 $$|{S}_{1}\rangle={|-{a}_{2}\alpha \rangle}_{0H}{|0\rangle}_{0V}\otimes [(1+\frac{{g}^{2}{{a}_{2}}^{2}|\alpha {|}^{2}}{2})(1-\frac{{g}^{2}|\alpha {|}^{2}}{2}){|0,0\rangle}_{1}{|0,0\rangle}_{2}+ig\alpha {a}_{1}{|1,0\rangle}_{1}{|0,1\rangle}_{2}].$$

Note that a similar output would be obtained by setting *b*_1_ = *a*_2_, *A* = 1 and *ϕ* = *π*.

Bob can not directly measure the state |*ψ*_*A*_〉 from the current system state, but he can perfectly recover it by feeding the three signals into an additional nonlinear crystal, namely BBO_3_ (compensating for path differences). In an experimental realization, this is most simply done by feeding the third BBO with the same pump laser used for the first and second BBO instead of using a new laser, since the use of two different sources requires dealing with phase fluctuations between them. However, it is theoretically possible to consider independent laser sources with no relative phase fluctuations, and we consider that case to show the minimum required number of transmitted resources between Alice and Bob in our protocol. This can be done because SPDC leaves coherent pump states invariant up to order *g* ^3^ in probability, and consequently BBO emissions are still indistinguishable when different pumps are used.

It could be argued that part of the information of the sent state |*ψ*_*A*_〉 is in the coherent laser state that Bob needs to pump BBO_3_ (which is equal to the pump state right after BBO_2_). However, such information is instead contained in the intensity ratio between the single photons and Bob’s coherent laser, such that Bob’s required coherent laser state can be fixed as |*α*_2_〉 for all possible states |*ψ*_*A*_〉 (up to a zero-measure set) by allowing Alice to regulate the initial intensity of her coherent laser accordingly. Here, *α*_2_ is a constant agreed beforehand with Bob. Alice sets α = *α*_2_/*a*_2_ in her initial pump beam preparation. This way, Bob would recover |*ψ*_*A*_〉 afterwards by pumping BBO_3_ with a state agreed beforehand that is independent of |*ψ*_*A*_〉. Thus, the only intensity that will depend on the state encoded by Alice will be the average number of single photons travelling through the decoherence channel.

**Figure 2:**
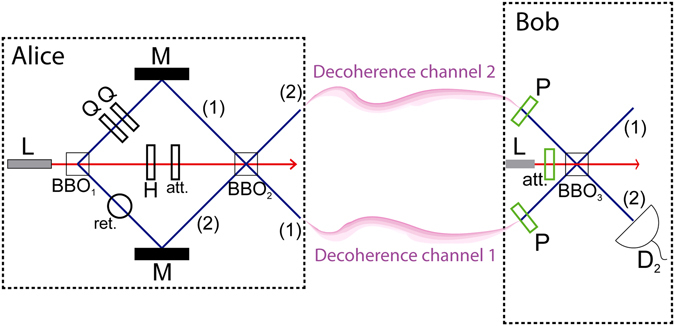
Alice wants to send an arbitrary qubit |*ψ*_*A*_〉 = *a*_1_|*H*〉 + *a*_2_|*V*〉 to Bob. She writes and encodes her qubit using our previous quantum adder setup. She then sets her H waveplate, attenuator and retarder so that b_1_ = 1, *A* = *a*_2_ and *ϕ* = *π*, such that all single photons after BBO_2_ have |*ψ*_*A*_〉 independent polarizations. Afterwards, single photons are sent through decoherence channels and received by Bob. He projects them into their desired constant polarization, and attenuates his identical coherent laser to account for expected losses in the single photons. Finally, he feeds BBO_3_ with those three signals, obtaining |*ψ*_*A*_〉 in path (1) with a high success probability.

Before letting Bob reconstruct |*ψ*_*A*_〉, we send our current state through decoherence channels affecting the single-photon paths. We show the behavior of our system under depolarizing and dephasing channels. The whole setup is depicted in Fig. 2. It is worth noting that the required pump beam for BBO_3_ will be slightly different after considering the channels, but will be independent of |*ψ*_*A*_〉 nevertheless.

## Depolarizing channel

Let us compute the effect of the depolarizing channel on our setup and compare it with the success probability of both a direct transmission of the unencoded quantum state through the same noisy channel, and a similar setup using a distilled quantum teleportation protocol. The success probability is the ratio between the reconstructed states which actually encode the original qubit sent by Alice and the total number of states reconstructed by Bob.

To calculate the decoherence caused by a depolarizing channel, we consider a three level system in path *x* (1 or 2), formed by any linear combination of the states $\{{|\mathrm{0,}0\rangle }_{x},{|\mathrm{1,}0\rangle }_{x},{|\mathrm{0,}1\rangle }_{x}\}$. For simplicity, we will temporarily refer to these states as $\{{|0\rangle }_{x},{|H\rangle }_{x},{|V\rangle }_{x}\}$. The state |0_*x*_〉 can not be dephased or flipped into |*H*_*x*_〉 or |*V*_*x*_〉 by depolarizing or dephasing decoherence channels (though the other states can dephase with respect to it).

Thus, for an initial density matrix

6 $${\rho }_{i}=(\begin{array}{ccc}a & {x}_{1} & {x}_{2}\\ {x}_{1}^{\ast } & b & {x}_{3}\\ {x}_{2}^{\ast } & {x}_{3}^{\ast } & c\end{array})$$

written in our $\{{|0\rangle }_{x},{|H\rangle }_{x},{|V\rangle }_{x}\}$ basis, the density matrix after the depolarizing channel would be

7 $${\rho }_{f}=(\begin{array}{ccc}a & \sqrt{1-p}{x}_{1} & \sqrt{1-p}{x}_{2}\\ \sqrt{1-p}{x}_{1}^{\ast } & \frac{(b+c)p}{2}+(1-p)b & (1-p){x}_{3}\\ \sqrt{1-p}{x}_{2}^{\ast } & (1-p){x}_{3}^{\ast } & \frac{(b+c)p}{2}+(1-p)c\end{array})\,.$$

Here, *p* = 1 − *e* ^*−γL/c*^ is a parameter ranging between 0 and 1 that measures depolarization, where *γ* is the depolarizing parameter, *L* the channel distance and *c* the speed of light. Let us now provide the Kraus operator version of this transformation. For that end, we require the following 5 Kraus operators:

8 $$\begin{array}{c}{E}_{0}=(\begin{array}{ccc}1 & 0 & 0\\ 0 & \sqrt{1-p} & 0\\ 0 & 0 & \sqrt{1-p}\end{array})\,,\quad {E}_{1}=(\begin{array}{ccc}0 & 0 & 0\\ 0 & \sqrt{p/4} & 0\\ 0 & 0 & \sqrt{p/4}\end{array})\,,\quad {E}_{2}=(\begin{array}{ccc}0 & 0 & 0\\ 0 & 0 & \sqrt{p/4}\\ 0 & \sqrt{p/4} & 0\end{array})\,,\\ \quad \quad \quad \,{E}_{3}=(\begin{array}{ccc}0 & 0 & 0\\ 0 & 0 & -\sqrt{p/4}\\ 0 & \sqrt{p/4} & 0\end{array})\,,\quad {E}_{4}=(\begin{array}{ccc}0 & 0 & 0\\ 0 & \sqrt{p/4} & 0\\ 0 & 0 & -\sqrt{p/4}\end{array})\,,\end{array}$$

fulfilling the gauge condition ${\rho }_{f}={\sum }_{n=0}^{4}{E}_{n}{\rho }_{i}{{E}_{n}}^{\dagger }$, so they define a completely positive trace preserving map. Moreover, we have checked that this is the minimum number of Kraus operators required to describe this transformation by constructing the Choi matrix and analyzing its spectrum^32^.

The density matrix output by this transformation satisfies a set of conditions that are expected from a decohering three level system, in which the first state (|0〉_*x*_) is invariant:

- The trace is preserved and the map completely positive.
- The density matrix is invariant if *p* = 0.
- The first diagonal term never changes.
- The second and third diagonal terms are equal when *p* = 1.
- All the coherences vanish when *p* = 1, but those between {|*H*〉_*x*_, |*V*〉_*x*_} vanish quadratically faster than those between {|0〉_*x*_, |*H*〉_*x*_} and {|0〉_*x*_, |*V*〉_*x*_}, as we vary from *p* = 0 to *p* = 1.

This depolarizing transformation is applied on beams 1 and 2 of the state in Eq. (5). Note that the single photons have |*ψ*_*A*_〉 independent polarization. After the depolarizing channel, to correct the state as much as possible, we project path (1) and path (2) into horizontal and vertical polarization, respectively. Bob aligns the single photons into BBO_3_ and pumps it with a (horizontally polarized) coherent laser $|{(1-\frac{p}{2})}^{2}{\alpha }_{2}\rangle$, which is identical to the coherent state right after BBO_2_, but attenuated by a factor of ${(1-\frac{p}{2})}^{2}$. This attenuation is necessary to compensate for the intensity loss in the single photons when projecting them with the polarizers. A percentual error in the estimation of ${(1-\frac{p}{2})}^{2}$ for the pump beam attenuation would cause the same percentual error in the vertical-polarization photon population after the SPDC process, so small errors in this estimation do not significantly affect the state reconstruction. After the attenuation and BBO_3_, we calculate the success probability of our protocol, that is, the probability for path-(1) polarization states to be measured as |*ψ*_*A*_〉, and obtain

9 $${P}_{{\rm{P}}{\rm{S}}{\rm{T}}}=1-\frac{1}{4}(\frac{p/2}{1-p/2})(1-\,\cos \,4\theta ),$$

where we have parametrized *a*_2_ = sin *θ*.

**Figure 3:**
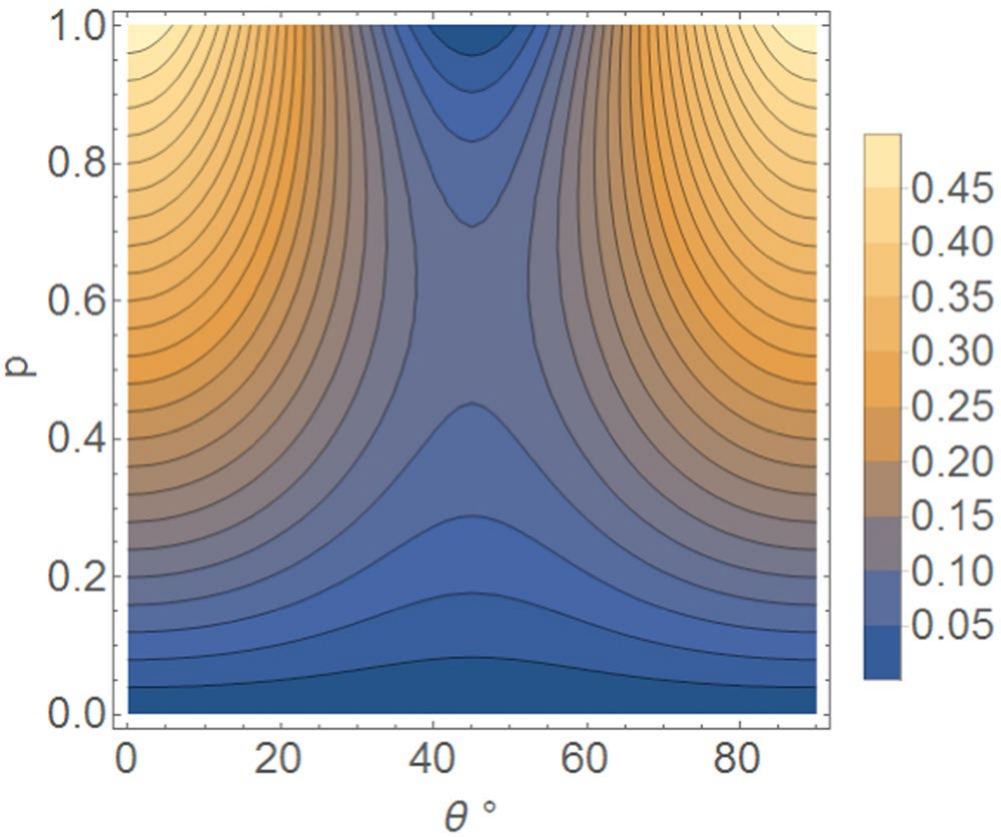
Success probability enhancement (difference) of our protocol sending a qubit |*ψ*_*A*_〉 = *e* ^*iϕ*^cos*θ|H*〉 + sin*θ|V*〉 through a depolarization channel, with respect to direct transmission. The degree of depolarization is parametrized by *p*, and *θ* is plotted between 0° and 90°. The plot is symmetric respect to *θ* = 0°.

If |*ψ*_*A*_〉 is sent straightforwardly through the depolarizing channel, the success probability is ${P}_{{\rm{straight}}}=1-\frac{p}{2}$. Our protocol has advantage in any point {*p*, *θ*} up to a zero-measure set (*p* = 0, where both have perfect success, and {*p* = 1, *θ* = 45°}, where both have success probability 0.5). The enhancement of our protocol is shown in Fig. 3.

An intuitive way to understand why our protocol protects the qubit information against depolarization is that the vertically-polarized amplitude of our initial qubit is not present when the single photons traverse the depolarizing channel. Instead, that population information is encoded in the average number of single photons traversing the channel: a higher number of single photons means that the initial encoded state was closer to being a horizontally polarized state, and vice versa. The vertical-polarization amplitude is afterwards recovered with the third BBO, which “creates” vertical photons where the single-photon state |0〉 was. This way, the reconstructed vertical component shows decoherence with respect to the horizontal component that is equivalent to the channel’s relative decoherence between |0〉 and |*H*〉, instead of the channel’s relative decoherence between |*H*〉 and |*V*〉, which would be higher. This reasoning also explains why the protocol works best for initial states that are closer to being purely horizontal or purely vertical: since the two components are separated while the photons traverse the channel, it is possible to clean the signal by using polarizers right after the depolarizing channel, so that pure horizontal and pure vertical initial states are reconstructed perfectly.

Decoherence protected state transfer can also be realized with quantum distillation. Alice prepares *N* Bell pairs and sends one party of each pair through the same noisy channel to Bob. This way, they share pairs of states ${W}_{{F}_{0}}$ that resemble Bell pairs, with probability ${F}_{0}=1-\frac{3p}{4}$. Quantum distillation consists in using LOCC between the two parties to reconstruct *m* (*m* < *N*) pairs of states *W*_*F*_, which resemble Bell pairs with probability *F* (*F* > *F*_0_). These states are then used in a teleportation protocol to transfer an arbitrary qubit between the two parties, so that *F* is proportional to the success probability sending the qubit. We assume perfect classical communication.

Although there are several distillation protocols, we compare our proposal against the one with the best success probability without making use of additional pre-shared resources, within those proposed by Bennet *et al*.^12^. In this protocol, every two states ${W}_{{F}_{0}}$ are distilled into one state ${W}_{{F}_{1}}$, where ${F}_{1}=1-\frac{2}{3}\mathrm{(1}-{F}_{0})$ to the lowest order in (1 − *F*_0_), and there is a $\frac{2}{3}\sqrt{1-{F}_{0}}$ probability for this process to fail, when the pair of states ${W}_{{F}_{0}}$ are discarded. To same order, *k* iterations of this procedure produce states ${W}_{{F}_{k}}$ with

10 $${F}_{k}=1-{(\frac{2}{3})}^{k}(1-{F}_{0}).$$

**Figure 4:**
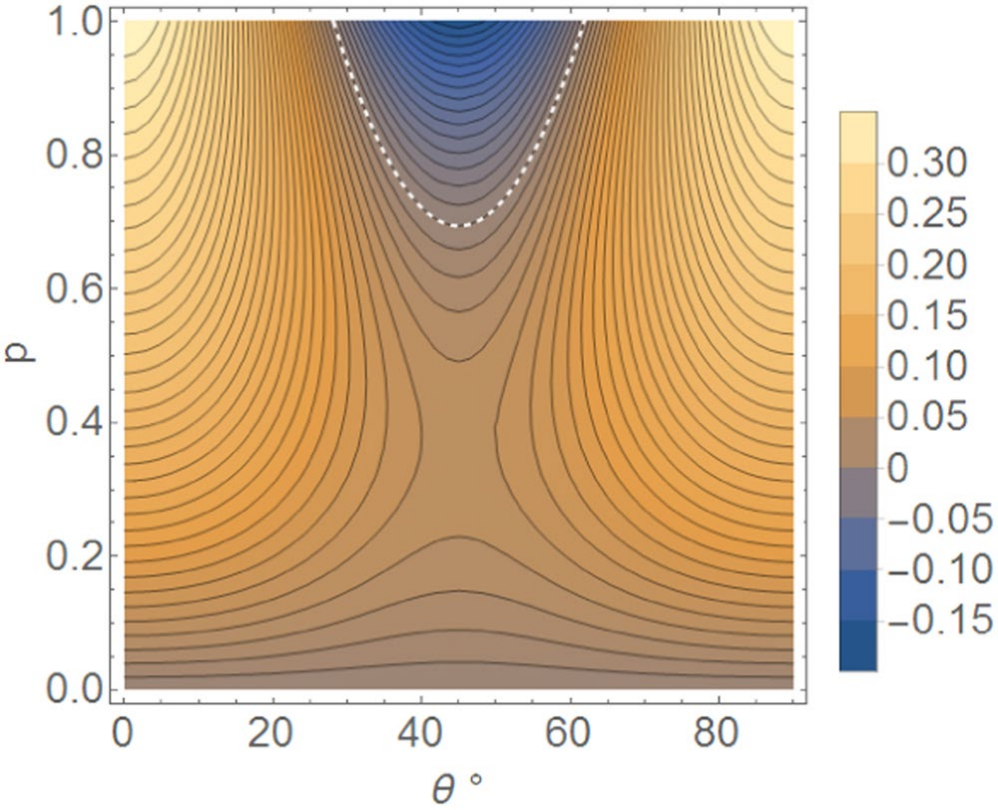
Success probability enhancement (difference) of our protocol in sending a qubit |*ψ*_*A*_〉 = *e* ^*iϕ*^cos*θ|H*〉 + sin*θ|V*〉 through a depolarization channel, with respect to distilled quantum teleportation protocol^12^. The region above the dashed line is the only region where the latter has higher success probability. The degree of depolarization is parametrized by *p*, and *θ* is plotted between 0° and 90°. The plot is symmetric respect to *θ* = 0°.

In both, our protected state transfer protocol and quantum teleportation with *k* distillation iterations, the ratio between the expected number of states reconstructed by Bob and the number of states sent by Alice is a measure of the number of resources used. In distilled teleportation, it is $\frac{1}{{2}^{k}}(1-\frac{2}{3}\frac{1}{1-\sqrt{\mathrm{2/3}}}(1-{(\sqrt{\mathrm{2/3}})}^{k})\sqrt{1-{F}_{0}})$, whereas in our protocol it is ${(1-\frac{p}{2})}^{2}$. We set these two quantities to be equal, in order to use the same amount of resources in both protocols, before comparing their respective success probabilities sending the qubit, as shown in Fig. 4.

It is shown that, for *p* ≲ 0.69, our protocol has a better success probability than distilled quantum teleportation. Furthermore, when *p* is greater than this threshold, distilled quantum teleportation only holds an advantage for 28° ≲ *θ* ≲ 62°.

## State-reconstruction protocol

After BBO_2_, the state of the system is given by Eq. (6) in the main text, that is

11 $$|{S}_{1}\rangle={|-{a}_{2}\alpha \rangle}_{0H}{|0\rangle}_{0V}\otimes [(1+\frac{{g}^{2}{{a}_{2}}^{2}|\alpha {|}^{2}}{2})(1-\frac{{g}^{2}|\alpha {|}^{2}}{2}){|0,0\rangle}_{1}{|0,0\rangle}_{2}+ig\alpha {a}_{1}{|1,0\rangle}_{1}{|0,1\rangle}_{2}].$$

Alice has set the initial pump beam state such that *α* = *α*/*a*_2_, where *α*_2_ is a constant agreed beforehand with Bob. We now include the additional coherent laser in our system, which Bob will use to pump BBO_3_. The (coherent) state of this laser is set to $|{(1-\frac{p}{2})}^{2}{\alpha }_{2}\rangle$, where *p* is a parameter between 0 and 1 that measures the depolarization caused by the channel between Alice and Bob. We will use the same indexes used for the first laser, but adding a “ + ”. This way, the complete system right after BBO_2_ is

12 $$\begin{array}{c}|{S}_{1}\rangle={|-{a}_{2}\alpha \rangle}_{0H}{|0\rangle}_{0V}{|{(1-\frac{p}{2})}^{2}{\alpha }_{2}\rangle}_{0H+}{|0\rangle}_{0V+}\otimes [(1+\frac{{g}^{2}{{a}_{2}}^{2}|\alpha {|}^{2}}{2})\\ \quad \quad \times \,(1-\frac{{g}^{2}|\alpha {|}^{2}}{2}){|0,0\rangle}_{1}{|0,0\rangle}_{2}+ig\alpha {a}_{1}{|1,0\rangle}_{1}{|0,1\rangle}_{2}),\end{array}$$

which is equivalent to

13 $$\begin{array}{c}|{S}_{1}\rangle={|-{\alpha }_{2}\rangle}_{0H}{|0\rangle}_{0V}{|{(1-\frac{p}{2})}^{2}{\alpha }_{2}\rangle}_{0H+}{|0\rangle}_{0V+}\otimes [(1+\frac{{g}^{2}{{a}_{2}}^{2}|\alpha {|}^{2}}{2})\\ \quad \quad \times \,(1-\frac{{g}^{2}|\alpha {|}^{2}}{2}){|0,0\rangle}_{1}{|0,0\rangle}_{2}+ig{\alpha }_{2}\frac{{a}_{1}}{{a}_{2}}{|1,0\rangle}_{1}{|0,1\rangle}_{2}].\end{array}$$

If *p* = 0, that is, if there were no depolarization, after feeding BBO_3_ with the new pump laser and the single photon paths, Bob would obtain

14 $$\begin{array}{c}|{S}_{1}\rangle={|-{\alpha }_{2}\rangle}_{0H}{|0\rangle}_{0V}{|{(1-\frac{p}{2})}^{2}{\alpha }_{2}\rangle}_{0H+}{|0\rangle}_{0V+}\otimes [(1+\frac{{g}^{2}{{a}_{2}}^{2}|\alpha {|}^{2}}{2})\\ \quad \quad \times \,(1-\frac{{g}^{2}|\alpha {|}^{2}}{2}){|0,0\rangle}_{1}{|0,0\rangle}_{2}+ig{\alpha }_{2}\frac{{a}_{1}}{{a}_{2}}{|1,0\rangle}_{1}{|0,1\rangle}_{2}+ig{\alpha }_{2}{|0,1\rangle}_{1}{|0,1\rangle}_{2}],\end{array}$$

where *R* is a renormalization parameter. This system state is equivalent to

15 $$\begin{array}{c}|{S}_{1}\rangle={|-{\alpha }_{2}\rangle}_{0H}{|0\rangle}_{0V}{|{(1-\frac{p}{2})}^{2}{\alpha }_{2}\rangle}_{0H+}{|0\rangle}_{0V+}\otimes [(1+\frac{{g}^{2}{{a}_{2}}^{2}|\alpha {|}^{2}}{2})(1-\frac{{g}^{2}|\alpha {|}^{2}}{2})\\ \quad \quad \times \,{|0,0\rangle}_{1}{|0,0\rangle}_{2}+ig\frac{{\alpha }_{2}}{{a}_{2}}({a}_{1}{|1,0\rangle}_{1}+{a}_{2}{|0,1\rangle}_{1}){|0,1\rangle}_{2}],\end{array}$$

where we can notice that the state |*ψ*_*A*_〉 has been perfectly reconstructed in the single photon polarization of path (1). If *p* ≠ 0, this process will no longer be perfect, and when *p* = 1, the probability for the output to be measured as |*ψ*_*A*_〉 will be 0.5.

To obtain the success probability of this protocol for *p* ≠ 0, we write the system state in Eq. (13) as a density matrix and then apply the decoherence channel transformation on the single photons, for each one of the two paths. Afterwards, the BBO_3_ transformation is applied, and we then determine the probability for the single-photon polarization state in path (1) to be measured as |*ψ*_*A*_〉. This calculation is obtained as a function of *p* and *θ*, where the latter is a parametrization of *a*_2_.

## Dephasing channel

Let us also compare the behavior of our protocol against a dephasing channel. Using a parameter *β*_*x*_ between 0 and 1 to measure dephasing in single-photon path *x*, we note that the coherences between {|1, 0〉_*x*_, |0, 1〉_*x*_} vanish quadratically faster than those between {|0, 0〉_*x*_, |1, 0〉_*x*_} and between {|0, 0〉_*x*_, |0, 1〉_*x*_}. In our protocol, the coherences of the sent state are multiplied by a factor of $\sqrt{1-{\beta }_{1}}\sqrt{1-{\beta }_{2}}$, whereas in a straightforward transmission through path *x* they are multiplied by a factor of 1 − *β*_*x*_. The success probability of both protocols is thus the same when *β*_1_ = *β*_2_.

Indeed, in our protocol, after reconstructing the state with BBO_3_, the success probability is shown to be

16 $$1-\frac{1}{2}{\sin }^{2}\,(2\theta )(1-\sqrt{1-{\beta }_{1}}\sqrt{1-{\beta }_{2}}),$$

and the success probability for a polarization qubit straightforwardly sent through channel *x* is

17 $$1-\frac{1}{2}{\sin }^{2}\,(2\theta )\,{\beta }_{x}.$$

Summarizing, we propose a state transfer protocol with protection against depolarization. Our protocol shows success probability enhancement when compared against direct transmission and distilled quantum teleportation. Moreover, against symmetric dephasing channels, its success probability is not decreased with respect to direct transmission. Our setup is a practical application of a probabilistic quantum adder and is based on a spontaneous parametric down-conversion interferometer. This paves the way for novel long range quantum communication protocols through noisy channels and quantum information transfer.
